# Automated Brief Hospital Course Summarization in Cardiac Surgery Using a Lightweight Large Language Model–Based Framework: Development and Evaluation Study on the Medical Information Mart for Intensive Care-IV

**DOI:** 10.2196/90870

**Published:** 2026-09-24

**Authors:** Xiaoyuan Gao, Yang Wang, Zixing Wang, Jing Yuan, Shengkang Huang, Xu-Yao Zhang, Zhaohong Sun, Yun Xing, Yiyang Liu, Xintong Wu, Zhan Hu, Wei Zhao

**Affiliations:** 1 Chinese Academy of Medical Sciences & Peking Union Medical College Beijing China; 2 Department of Information Center, Fuwai Hospital, Chinese Academy of Medical Sciences and Peking Union Medical College Beijing China; 3 Information Center, First Hospital of Tsinghua University Beijing China; 4 Department of Cardiac Surgery, Fuwai Hospital, National Center for Cardiovascular Diseases Beijing China; 5 Institute of Automation of Chinese Academy of Sciences Beijing China; 6 School of Artificial Intelligence, University of Chinese Academy of Sciences Beijing China; 7 Statistical Information Center of the National Health Commission Beijing China

**Keywords:** automated summarization, clinician burnout, large language models, lightweight, natural language processing, patient discharge summaries, privacy-preserving

## Abstract

**Background:**

Physician documentation requirements are a known contributor to clinician burnout, with the manual creation of brief hospital course (BHC) summaries being particularly time-consuming. Automating BHC summarization may mitigate this workload and reduce documentation errors. However, current natural language processing (NLP) methods are often limited to single-document inputs, and large language models (LLMs) face privacy and deployment challenges. Furthermore, existing methods often require manual information extraction and struggle to maintain temporal accuracy.

**Objective:**

We developed and evaluated LiteMedDoc, a lightweight, locally deployable LLM-based framework to automatically generate BHC summaries without model fine-tuning. Our objective was to determine whether clinically useful clinical summaries could be generated under strict privacy and resource constraints, making automated summarization feasible in real-world hospital environments.

**Methods:**

LiteMedDoc is a modular pipeline built upon an 8-billion-parameter open-source LLM (Llama 3.1). It features 3 specialized modules: a static dynamic information hierarchy module to condense multisource inputs and structure clinical events chronologically; a similar document retrieval-augmented generation module that retrieves contextually relevant prior case summaries; and a self-adaptive feedback optimization module used offline for prompt optimization. All processing was performed locally without any model fine-tuning. We evaluated the framework on a retrospective cohort of 4538 coronary artery bypass grafting (CABG) surgery cases from the Medical Information Mart for Intensive Care (MIMIC)-IV database. A held-out test set of 403 cases was used to generate BHC summaries. The model-generated summaries were compared to reference BHCs using 8 standard NLP metrics covering lexical overlap (BLEU-4 [Bilingual Evaluation Understudy-4 gram] and ROUGE [Recall-Oriented Understudy for Gisting Evaluation]), semantic similarity (BERTScore and METEOR [Metric for Evaluation of Translation With Explicit Ordering]), and clinical relevance (AlignScore [Alignment Score] and MEDCON [Medical Concept Overlap]). Additionally, 15 cardiac surgeons conducted a clinical evaluation of a sample of model-generated summaries, rating them on completeness, correctness, readability, conciseness, and global quality using a 5-point Likert scale.

**Results:**

Without any model training, LiteMedDoc achieved strong performance across individual automated metrics, nearly matching a fine-tuned model and exceeding a 70-billion-parameter model on all metrics. Additionally, in a within-database cross-domain evaluation on lobectomy cases, the framework maintained encouraging performance after prompt adaptation and outperformed both the base model and the CABG-fine-tuned model. Surgeons rated the AI-generated summaries above the prespecified acceptability threshold across all domains (mean scores ≥3.0), specifically praising their structure and conciseness.

**Conclusions:**

By integrating 3 specialized modules, the proposed framework offers a practical, locally deployable solution for clinician-in-the-loop BHC draft generation under privacy and resource constraints, and holds promise for improving documentation efficiency and enhancing information continuity during care transitions.

## Introduction

The burden of clinical documentation is a significant factor in physician burnout [1-3], with electronic health record (EHR) documentation and desk work consuming nearly 2 additional hours for every hour of direct patient care [4]. Within this context, the brief hospital course (BHC), a vital narrative in the discharge summary synthesizing key events and management decisions, is particularly burdensome. Clinicians must review the entire inpatient record to write the BHC, since daily progress notes cannot be simply copied forward [5]. While a high-quality BHC is essential for continuity of care and patient safety [6], its manual creation is both time-consuming [7] and error-prone [8]. This process carries the risk of omissions and inaccuracies, which can lead to hospital readmissions or medication errors [9]. Consequently, there is a critical need for an automated tool capable of generating patient discharge summaries rapidly and accurately.

While automating BHC writing offers a promising solution [10,11], prior computational approaches have been proven inadequate. Specifically, conventional natural language processing (NLP) techniques are ill-suited for BHC generation as they often frame the task as single-document summarization [12,13]. Instead, creating a BHC necessitates the integration of heterogeneous data sources, such as daily notes, laboratory results, and imaging reports, combined with complex clinical reasoning [14]. The inadequacy of single-source methods is underscored by evidence that up to 39% of discharge summary content originates from external documents or clinician inference [15], content that such single-document-based approaches inherently fail to capture.

The emergence of large language models (LLMs) offers new opportunities for clinical document summarization [16,17]. However, a critical conflict between the strict data privacy standards of health care and the computational demands of high-performance models impedes real-world application. Closed-source LLMs (such as ChatGPT) can generate discharge summaries of comparable quality to physicians [18] while saving time [19], but relying on external servers raises insurmountable data privacy and security concerns in clinical environments [20]. Conversely, open-source LLMs allow on-premises deployment but typically lack the capability to handle complex clinical synthesis without extensive, resource-heavy fine-tuning [21].

Meanwhile, standardized benchmarks for procedure-specific BHC summarization remain limited. A closely related community benchmark, the “Discharge Me!” shared task at the Biomedical Natural Language Processing (BioNLP) Workshop, colocated with the Annual Meeting of the Association for Computational Linguistics, targets generation of the BHC and Discharge Instructions sections using curated subsets derived from Medical Information Mart for Intensive Care (MIMIC)-IV-Note and MIMIC-IV-Emergency Department [22]. However, its cohort composition, inputs, and evaluation setting differ from the present coronary artery bypass grafting (CABG)–only inpatient cardiac-surgery cohort and our multisource aggregation framework. Accordingly, this study provides an initial task-specific reference point for privacy-preserving, procedure-focused inpatient BHC summarization.‌

Beyond these resource and architectural challenges, a fundamental limitation persists: existing approaches often struggle with core clinical requirements, such as accurate temporal reasoning [23]. Since clinical summaries must adhere to a strict chronological order, current tools still demand manual key information extraction by clinicians to ensure coherence [24]. These multifaceted challenges call for a solution that is strict on privacy, resource-efficient, and capable of fully automated, clinically coherent summarization directly from raw EHR data.

To bridge this gap, we developed LiteMedDoc, a lightweight, locally deployable framework for medical document summarization. LiteMedDoc systematically addresses prior limitations through 3 plug-and-play modules designed to handle long multisource inputs, inject domain knowledge, and ensure factual consistency. This design empowers smaller, open-source LLMs to generate high-quality BHC summaries under real-world constraints, without the need for fine-tuning or manual data extraction. We evaluated the framework’s performance using both automated metrics and expert clinical review, assessing its performance against physician-written summaries and other modeling approaches.

## Methods

### Data Source and Processing

This study used the MIMIC-IV database [25], a publicly available repository containing deidentified EHR from Beth Israel Deaconess Medical Center (2008-2019). We specifically targeted inpatient admissions involving CABG surgery, identified via relevant *International Classification of Disease* (*ICD)* procedure codes. This selection process yielded a total of 5670 distinct admissions.

To establish a reference target for summarization evaluation, we extracted the BHC section from the corresponding discharge summaries. We excluded admissions with missing discharge summary text (n=41), those with a missing/empty BHC section after section parsing (n=445), and those with BHC text shorter than 150 words and/or clearly inconsistent with a surgical BHC narrative (n=646). The physician-written BHCs in this study served not only as evaluation targets but also, for the nontest subset, as retrieval exemplars in the similar document retrieval-augmented generation (SD-RAG) knowledge base. During cohort curation, very short or nonsurgical BHCs were frequently insufficiently informative, lacked key perioperative trajectory content, or did not reflect a coherent surgical hospital-course narrative. We therefore excluded such cases to maintain a minimally informative reference set for both retrieval and evaluation. This process resulted in 4538 valid reference summaries with an average length of 310 (SD 122; range 151-747) words.

For each admission, we aggregated a comprehensive input context derived from diverse EHR source documents, including demographics, diagnoses, laboratory results, imaging studies, procedures, prescriptions, discharge disposition, and other relevant clinical data [26]. The resulting compiled raw input was extensive, averaging 3937 (SD 1026; range 2014-6615) words per case.

The processed dataset was partitioned to support both the retrieval component and model evaluation. A random sample of 403 summaries (approximately 10%) was sequestered as a stand-alone test set for model evaluation. The remaining 4135 summaries were indexed to form a reference corpus (knowledge base), which serves as the retrieval source for the SD-RAG module described below. The 400 cases used for offline prompt optimization in the self-adaptive feedback optimization (SFO) module were sampled from this reference corpus. None of the 403 held-out test cases was included in any prompt-optimization batch.

### Ethical Considerations

This study used the publicly available, fully deidentified MIMIC-IV database. The creation and sharing of MIMIC-IV was reviewed by the Institutional Review Board of Beth Israel Deaconess Medical Center (2001-P-001699/14), which granted a waiver of informed consent and approved data sharing. Our analysis constituted secondary research on anonymized data and was therefore exempt from additional ethics review and informed consent. Access was obtained via PhysioNet after completing required human-subjects training and signing the data use agreement.

### Framework (LiteMedDoc)

#### Overview

As illustrated in Figure 1, the LiteMedDoc framework combines case-level inference modules with an offline prompt-optimization component. The framework integrates a locally deployed, pretrained LLM (Llama 3.1, 8B parameters, Meta AI, July 2024 release) [27] with 3 specialized “plug-and-play” modules: the static dynamic information hierarchy (SDIH) module, the SD-RAG module, and the SFO module. During routine inference, the SDIH module condenses and structures the multisource patient record, and the SD-RAG module supplements generation with clinically similar prior cases. Separately, the SFO module is used offline to optimize and update the prompting strategy on previous cases. In routine deployment, the system generates a single clinician-editable draft per patient using the most recently optimized prompt. The system was implemented on a single NVIDIA L20 GPU with 48 GB of VRAM, with an average end-to-end BHC generation time of 7.34 seconds per case.

**Figure 1:**
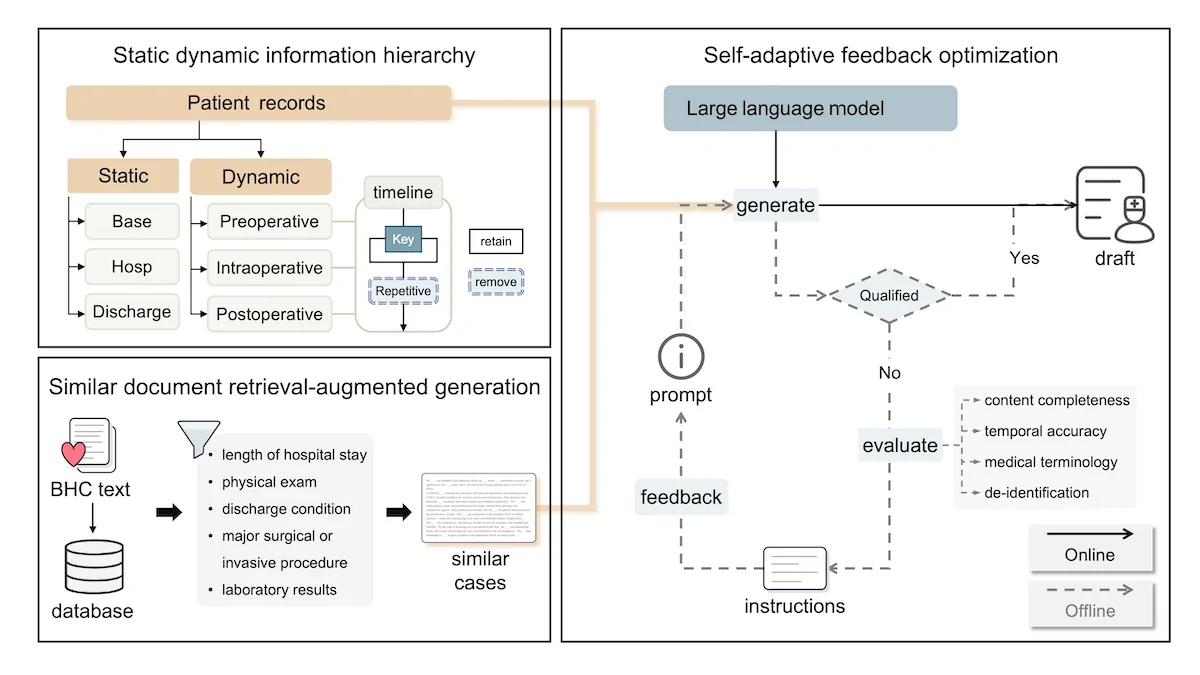
LiteMedDoc summarization framework overview. These specialized modules operate sequentially to assist an 8B large language model in generating the brief hospital course (BHC). In Module 3, the dashed loop denotes the offline prompt-optimization stage of the self-adaptive feedback optimization module.

#### Module 1: SDIH

To address the challenge of processing lengthy multisource EHR data, the SDIH module first transforms unstructured patient records into a structured format. It then conceptually partitions clinical data into static and dynamic components using rule-based parsing.

Static information refers to time-invariant elements, such as demographics and diagnoses. To facilitate precise information extraction, these data are further categorized by care phases: baseline background (base), hospitalization course (hosp), and discharge information (discharge).

Dynamic data, encompassing time-evolving clinical events such as laboratory results, prescriptions, and procedure events, were first aggregated from heterogeneous EHR sources and unified into a date-centric temporal structure. A rule-based algorithm subsequently classified clinical phases using surgical dates as anchor points. Specifically, the timeline was segmented into preoperative, intraoperative, and postoperative periods relative to the procedure date. To manage data volume, the module implemented a state-transition compression mechanism. Instead of retaining repetitive daily logs, the system filtered for clinical turning points by explicitly recording only the initiation or discontinuation of medications and the onset or normalization of abnormalities. This approach effectively condenses temporal density while preserving the clinical trajectory, mirroring the physician’s cognitive process of prioritizing pivotal developments over redundant routine data.

#### Module 2: SD-RAG

Following structural organization, the SD-RAG module was designed to improve factual completeness and mitigate hallucinations through a 3-step retrieval-augmented generation approach [28]. First, we constructed a vector database from our reference corpus (n=4135) by converting each summary into a vector embedding using a MiniLM sentence encoder [29] and indexing the resulting embeddings via Facebook AI Similarity Search (FAISS). Second, to ensure clinical relevance, the module uses a random forest–based feature importance analysis [30] to identify key patient features for case matching (see Multimedia Appendix 1 for more details). Finally, for a given patient, the module autonomously retrieves similar historical cases according to cosine similarity matching.

These retrieved summaries provide the LLM with “few-shot” examples and contextual grounding. Unlike using static medical knowledge bases, our case library can continuously grow with new cases, allowing the model to adapt to evolving clinical knowledge without retraining.

#### Module 3: SFO

The SFO module was implemented primarily as an offline prompt-optimization mechanism. During development, maintenance updates, or transfer to a new clinical domain, previous cases were processed in prespecified evaluation batches using the current prompt configuration. The resulting summaries were assessed across four prespecified quality dimensions: (1) content completeness (coverage of all important input information); (2) temporal accuracy (correct chronological order and day counts); (3) terminology use (appropriate and consistent medical terminology); and (4) deidentification (absence of any personal or institutional identifiers). These 4 dimensions were used to synthesize feedback for prompt revision, whereas the decision on whether to adopt a revised prompt configuration was based on the 8 automated metrics. When recurrent deficiencies were identified, feedback instructions were synthesized and used to revise the prompt, which was then carried forward to subsequent batches. Optimization continued until 3 consecutive batches failed to demonstrate sufficient improvement according to the predefined metric-based retention criterion. The retained prompt configuration was then used for subsequent batches and for routine inference. In routine deployment, LiteMedDoc generates a single clinician-editable draft per patient using the optimized prompt. Detailed technical procedures are provided in Multimedia Appendix 2.

In effect, the SFO module serves as an offline prompt-quality assurance mechanism that improved subsequent inference without modifying model parameters. This approach preserves the framework’s lightweight nature. Adapting the framework to a new clinical domain or documentation style, therefore, simply requires prompt or feedback rule updates, rather than model retraining.

### Prompt Design

#### Overview

Our prompting strategy was developed in collaboration with cardiac surgeons to ensure alignment with documentation standards. The strategy comprises 2 distinct components (examples are provided in Figure S1 and S2 in Multimedia Appendix 3).

#### Structure Prompt

It serves as a template, explicitly defining the required sections (admission context, preoperative preparation, surgical details, immediate postoperative recovery, subsequent progress, and final disposition) and information granularity.

#### Instruction Prompt

It defines stylistic guidelines, instructing the model to use concise professional language and adhere strictly to deidentification protocols.

### Models and Comparator Baselines

We benchmarked LiteMedDoc against multiple comparators to contextualize the effects of model family, model scale, domain adaptation, and deployment setting. The evaluated models included Mistral-7B-v0.3 [31] as a scale-matched open-source baseline; Llama 3.1-8B as the base backbone used in LiteMedDoc; Llama 3.1-70B as a larger-scale open-source comparator; Qwen3-14B [32] as an alternative open-source backbone; GPT-4o-mini [33] as a widely used closed-source reference model; and a supervised fine-tuning baseline built on the same Llama 3.1-8B backbone (Llama-8B-SFT). GPT-4o-mini was accessed through Azure OpenAI Service, following PhysioNet guidance for responsible use of GPT-family models with credentialed MIMIC data. This design allowed us to compare architecture at a similar scale, scaling effects within the same model family, domain-oriented performance, and performance relative to a commonly used closed-source model.

### Low-Rank Adaptation Supervised Fine-Tuning Baseline (Llama-8B-SFT)

To provide a parameter-efficient supervised fine-tuning comparator, we trained a Llama-8B-SFT baseline on the CABG cohort. Using the same split as the main benchmark, 4135 CABG cases were used for training, and 403 held-out CABG cases were used for testing.

Starting from the same Llama 3.1-8B backbone, supervised fine-tuning was implemented in LLaMA Factory using low-rank adaptation (LoRA) adapters while the backbone weights remained frozen. The model was trained to generate the reference BHC directly from the compiled patient input. The LoRA configuration used rank=8, alpha=16, dropout=0.05, target modules q_proj and v_proj, learning rate 2×10^–4^, per-device batch size 4, gradient accumulation steps 8, 3 training epochs, and bf16 mixed precision. The resulting model enhances factual completeness and mitigates hallucinations, denoted Llama-8B-SFT.

### Cross-Domain Evaluation Cohort (Lobectomy)

To assess cross-domain generalizability, we additionally extracted thoracic surgery admissions involving lung lobectomy from MIMIC-IV using *ICD* procedure codes. We applied the same discharge-summary parsing, BHC eligibility criteria, and cohort construction logic used for the CABG cohort.

This process yielded 696 lobectomy admissions with usable BHC references. Of these, 662 cases were indexed as the lobectomy retrieval corpus for the SD-RAG module, and 34 cases were reserved as a held-out lobectomy test set. For cross-domain comparison, we evaluated the untuned Llama 3.1-8B backbone, the CABG-specific Llama-8B-SFT model, and LiteMedDoc on this held-out lobectomy test set. LiteMedDoc was applied to this cohort with prompt wording adapted from CABG to lobectomy terminology. No additional model fine-tuning was performed on lobectomy data.

### Evaluation Method

To provide a comprehensive assessment of the generated summaries, we used a mixed methods approach combining automated metrics with expert physician reviews.

#### Automated Performance Metrics

##### Overview

We calculated 8 established metrics to evaluate performance across 3 linguistic and clinical dimensions. These automated metrics compare model outputs against physician-written reference summaries.

##### Lexical Overlap

BLEU-4 (Bilingual Evaluation Understudy-4 gram) [34] and ROUGE (Recall-Oriented Understudy for Gisting Evaluation), including ROUGE-1, ROUGE-2, and ROUGE-L [35], measure the word-level overlap between the model-generated summary and reference summary, indicating how much of the reference’s content is covered. Higher BLEU-4 or ROUGE scores suggest the summary captured more information from the reference text.

##### Semantic Similarity

BERTScore [36] and METEOR (Metric for Evaluation of Translation With Explicit Ordering) [37] assess similarity in meaning between the generated and reference summaries, going beyond exact word matches. These metrics use semantic embeddings or weighted overlaps to evaluate whether the model conveyed the same key ideas as the reference.

##### Clinical Accuracy

AlignScore (Alignment Score) [38] and MEDCON (Medical Concept Overlap) [39] evaluate factual consistency with the source record. AlignScore checks whether clinical facts stated in the summary align with details in the patient’s data. MEDCON measures the overlap of medical concepts (diagnoses, procedures, medications, etc) from the original record that appear in the summary. A higher MEDCON indicates the summary captured more of the important clinical entities from the case.

#### Expert Physician Evaluation

Complementing the automated analysis, a panel of 15 cardiac surgeons conducted a qualitative review. All reviewers were specialist cardiac surgeons practicing in tertiary hospitals, with 5-10 years of independent clinical experience.

A common set of 10 unique CABG cases was randomly sampled from the held-out test set, resulting in 150 paired evaluations (15 reviewers × 10 cases). For each case, reviewers were presented with the AI-generated summary alongside the corresponding physician-written note (reference note) and were asked to rate the AI-generated summary on a 5-point Likert scale across 5 dimensions: completeness, correctness, readability, conciseness, and global quality (questionnaire in Multimedia Appendix 4). Reviewers were not blinded to the source of the summaries. The AI-generated text was explicitly labeled to reflect the intended real-world workflow in which the model output is used as a clinician-editable draft that must be reviewed and corrected before finalization.

Interrater reliability across the 15 reviewers was assessed using the intraclass correlation coefficient (ICC), using a 2-way random-effects model for absolute agreement of mean ratings (ICC_2,k_). We report ICC estimates with 95% CIs for each evaluation domain.

Following prior work [40], the rating protocol prespecified a mean score of ≥3 before physician scoring as the operational threshold for baseline acceptability in a clinician-in-the-loop drafting workflow. This threshold was intended to indicate that a generated summary could serve as a usable draft for clinician review and edit, rather than a document suitable for unreviewed autonomous clinical use. Additionally, surgeons provided free-text qualitative feedback regarding the strengths and weaknesses of the generated texts.

#### Postoperative Day Safety Audit

Given the clinical importance of temporal accuracy, we prespecified a postoperative day (POD) safety audit. POD correctness was defined as agreement between the POD stated in a generated BHC and the POD derived from the patient’s structured timeline in the underlying record, based on the admission→surgery→discharge sequence. The audit evaluated only whether relative POD statements were consistent with the source timeline under deidentification constraints.

For the 10-case physician review sample, we manually checked POD consistency in both AI-generated summaries and physician reference notes. We additionally performed a cohort-level reaudit of all 4538 physician reference BHCs using an LLM-assisted auditing procedure that extracted surgery/discharge timing from the notes, compared it with the structured timeline, and flagged discrepancies for follow-up review. During the auditing pipeline, some model outputs failed the prespecified schema-based parsing step. These cases were manually inspected to find temporal-recognition errors. The final numerator for the cohort-level reaudit was calculated as the number of cases flagged by the automated auditor plus the additional inconsistencies identified by manual inspection of parsing failures.

## Results

### Model Performance Comparison Using Automated Metrics

We first evaluated LiteMedDoc against multiple baselines using 8 automated metrics on the held-out test set of 403 CABG cases (Table 1). Among untuned general-purpose models, performance varied widely. Mistral-7B-v0.3 and Qwen3-14B showed limited overlap with the reference BHCs (BLEU-4: 0.12 and 0.29, respectively), whereas Llama 3.1-70B and GPT-4o-mini achieved moderate gains but remained clearly below LiteMedDoc across all 8 metrics (eg, ROUGE-1: 40.79 and 39.82 vs 52.78; AlignScore: 21.94 and 23.76 vs 30.78). The CABG-specific Llama-8B-SFT baseline remained strongest on several individual metrics, including BLEU-4 (16.49), BERTScore (43.51), METEOR (35.00), AlignScore (44.38), and MEDCON (36.77).

**Table 1:** Performance of LiteMedDoc vs other models in brief hospital course summarization.

			BLEU-4^a^		ROUGE^b^-1		ROUGE-2		ROUGE-L		BERTScore		METEOR^c^		AlignScore^d^		MEDCON^e^
**Different base models**																	
	Mistral-7B-v0.3	0.12		14.97		1.77		7.04		12.07		11.59		23.43		11.42	
	Llama 3.1-8B	1.77		30.73		8.01		14.85		20.50		17.12		22.75		18.42	
	Llama 3.1-70B	5.95		40.79		12.07		21.26		34.17		26.21		21.94		23.29	
	Llama-8B-SFT	16.49		47.74		26.84		34.64		43.51		35.00		44.38		36.77	
	Qwen3-14B	0.29		23.95		2.90		10.42		15.46		18.07		24.25		15.77	
	GPT-4o-mini	4.89		39.82		10.66		18.65		32.34		24.65		23.76		22.03	
**LiteMedDoc ablations**																	
	+SDIH^f^ only	5.27		38.11		9.06		18.55		30.88		25.35		20.67		21.30	
	+SD-RAG^g^ only	1.71		31.01		8.26		15.77		20.54		17.23		23.50		18.47	
	+SDIH and SD-RAG	12.80		41.47		13.16		22.34		36.93		32.98		28.85		30.25	
	LiteMedDoc (all modules)	13.90		52.78		28.81		35.24		38.67		34.20		30.78		30.41	
**Cross-domain test** **(lobectomy)**																	
	Llama 3.1-8B	1.30		30.51		5.48		13.79		24.80		18.73		19.06		22.25	
	Llama-8B-SFT	2.47		23.73		10.65		15.30		23.45		16.37		20.56		20.08	
	LiteMedDoc	10.98		40.72		12.84		21.87		36.85		32.75		27.86		30.06	
**Task-relevant external model (external reference)**																	
	WisPerMed^h^	12.40		45.30		20.10		30.80		43.80		40.30		31.50		41.10	

^a^BLEU-4: Bilingual Evaluation Understudy-4 gram.

^b^ROUGE: Recall-Oriented Understudy for Gisting Evaluation.

^c^METEOR: Metric for Evaluation of Translation with Explicit Ordering.

^d^AlignScore: Alignment Score.

^e^MEDCON: Medical Concept Overlap.

^f^SDIH: static dynamic information hierarchy.

^g^SD-RAG: similar document retrieval-augmented generation.

^h^WisPerMed is reported solely as an external, task-relevant reference. Its values were published BHC results from the broader Medical Information Mart for Intensive Care-IV shared-task cohort rather than the coronary artery bypass grafting–only test set used in this study.

Our proposed LiteMedDoc framework demonstrated substantial efficacy without additional task-specific fine-tuning. Using the same 8B backbone, LiteMedDoc achieved the highest ROUGE scores among all evaluated systems (ROUGE-1=52.78; ROUGE-2=28.81; and ROUGE-L=35.24), exceeding the fine-tuned CABG model (ROUGE-1=47.74; ROUGE-2=26.84; and ROUGE-L=34.64) and GPT-4o-mini (ROUGE-1=39.82; ROUGE-2=10.66; and ROUGE-L=18.65).

For external context only, WisPerMed [41] reported strong semantic and clinically oriented scores, including BERTScore 43.80, METEOR 40.30, and MEDCON 41.10. Because these values were obtained on the broader MIMIC-IV BHC shared-task cohort rather than on the CABG-only test set used here, they are not directly comparable with the present results.

### Physician Evaluation of Generated Summaries

All 15 surgeons completed evaluations for the sampled summary pairs, yielding 150 case comparisons in total. Figure 2 summarizes the distribution of their ratings, and Table 2 presents interrater reliability and mean scores across the 5 evaluation domains. Interrater reliability, assessed using ICC_2,k_, yielded moderate point estimates across most domains. The associated 95% CIs were nonetheless wide, with lower bounds falling into the poor-to-fair range for global quality (0.28), conciseness (0.29), readability (0.34), and completeness (0.25). Mean ratings were 3.13 (SD 0.97) for global quality, 3.93 (SD 0.72) for conciseness, 3.75 (SD 0.79) for readability, 3.56 (SD 1.36) for correctness, and 3.79 (SD 1.03) for completeness. All mean scores were at or above 3.0, supporting baseline acceptability for a clinician-in-the-loop drafting workflow. However, the presence of 17 score-1 ratings and 18 score-2 ratings in the correctness domain indicated that some summaries contained potentially clinically relevant inaccuracies that could affect downstream care if left uncorrected. Most of these low ratings were concentrated in cases initially interpreted as POD date errors, accounting for 14 of the 17 score-1 ratings and 12 of the 18 score-2 ratings. The remaining low ratings were associated with a small number of inaccuracies or simplifications in perioperative details, such as standardizing specific medication wording (eg, converting “IV amio and lopressor” to “amiodarone”).

**Figure 2:**
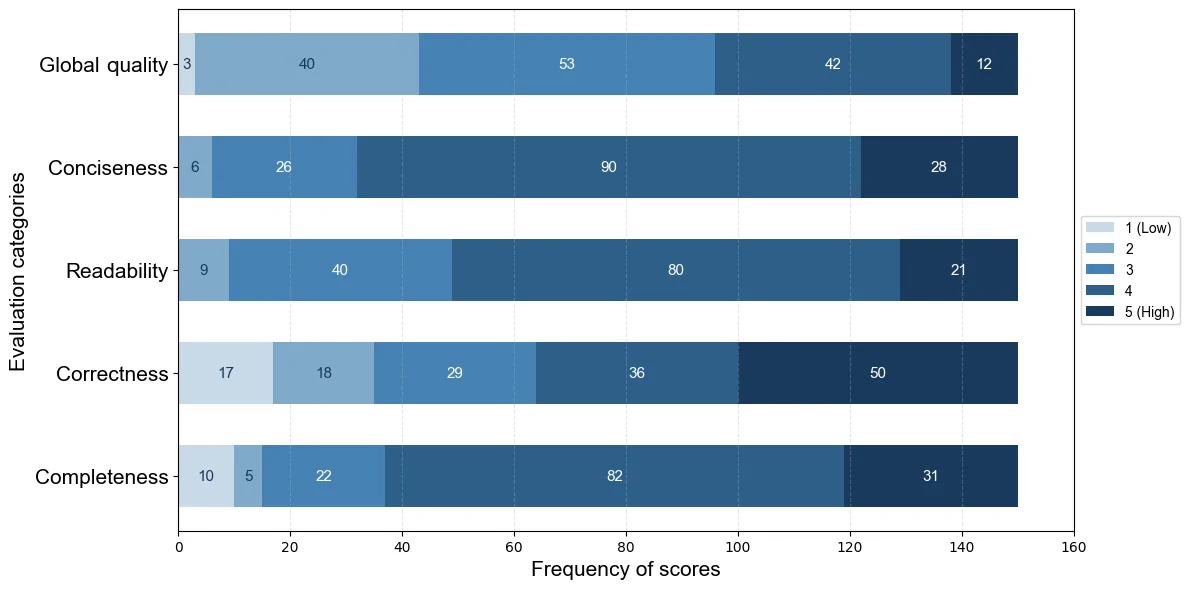
The distribution of 15 surgeons’ evaluations for 10 summary pairs. This stacked bar chart illustrates the frequency distribution of scores (1=low to 5=high) across the 5 evaluation categories.

**Table 2:** Interrater reliability and mean physician ratings for AI-generated summaries across 5 evaluation domains.

	ICC_2,k_^a^ (95% CI)	Rating, mean (SD)
Global quality	0.62 (0.28-0.88)	3.13 (0.97)
Conciseness	0.64 (0.29-0.89)	3.93 (0.72)
Readability	0.66 (0.34-0.89)	3.75 (0.79)
Correctness	0.77 (0.52-0.93)	3.56 (1.36)
Completeness	0.62 (0.25-0.88)	3.79 (1.03)

^a^ICC: intraclass correlation coefficient.

Because low correctness ratings were concentrated in cases with suspected discrepancies related to POD, we further examined temporal accuracy using the POD safety audit described in the Methods. In the 10-case clinician review sample, no POD discrepancies were identified in the AI-generated summaries, and manual review identified no errors or omissions in key inpatient milestones, including awakening status, recovery trajectory, and extubation timing. In contrast, POD discrepancies were identified in 4 of 10 physician reference notes. We then performed an expanded POD-specific cohort-level LLM-assisted reaudit of the 4538 physician reference BHCs. This reaudit flagged 288 (6.35%) of 4538 reference notes as containing potential POD inconsistencies after targeted follow-up review. A total of 286 flagged entries originated from automated audit, while 2 were identified through manual inspection of parsing failures. Because this estimate was derived from automated screening rather than exhaustive human adjudication, it should be regarded as a conservative lower bound indicating that the physician reference notes were not uniformly error-free, rather than as an exact population error rate.

Qualitative feedback highlighted that the AI-generated summaries were often more logically structured than physician-written notes, effectively organizing complex hospital courses by day or phase. Reviewers specifically appreciated the reduced extraneous verbosity typical of EHR notes. Some limitations were also noted, including occasionally stiff phrasing and oversimplification of nuanced clinical details due to brevity. Nonetheless, because discharge summaries may inform subsequent referral and follow-up care, these findings further support the need for physician review before finalization.

### Health Care Context Condensation and Input Length Effects

The efficiency of LiteMedDoc is largely driven by the SDIH module’s ability to condense lengthy clinical contexts. As illustrated in Figure 3, raw input texts typically ranged from 3000 to 6000 words (with some outliers beyond 6000 words). The SDIH module achieved a compression rate of approximately 50% or more, clustering processed inputs between roughly 1800 and 2300 word cases. By filtering redundant data (eg, routine vitals) while preserving critical events (eg, new-onset atrial fibrillation), the module transformed unstructured data into concise chronological segments (Figure 4), enabling the LLM to focus on core clinical content.

We further analyzed LiteMedDoc’s robustness across varying input lengths (Figure 5). The 403 test cases were grouped as short, medium, and long records. Performance was strongest for short and medium inputs and declined in the long-input group across all automated metrics. For example, BLEU-4 decreased from 21.08 (short) and 14.02 (medium) to 5.94 (long), ROUGE-2 from 32.48 and 29.87 to 11.05, and BERTScore from 45.53 and 39.60 to 29.10.

Long records typically represent more complex clinical courses or prolonged hospital stays (eg, multiple complications or prolonged intensive care unit care), making concise condensation more difficult. Nonetheless, LiteMedDoc retained nontrivial source-grounded performance even in long cases (AlignScore 25.72; MEDCON 22.85). In practice, extremely long records might be handled by further segmenting the input into subsections for separate summarization—an approach for future exploration.

**Figure 3:**
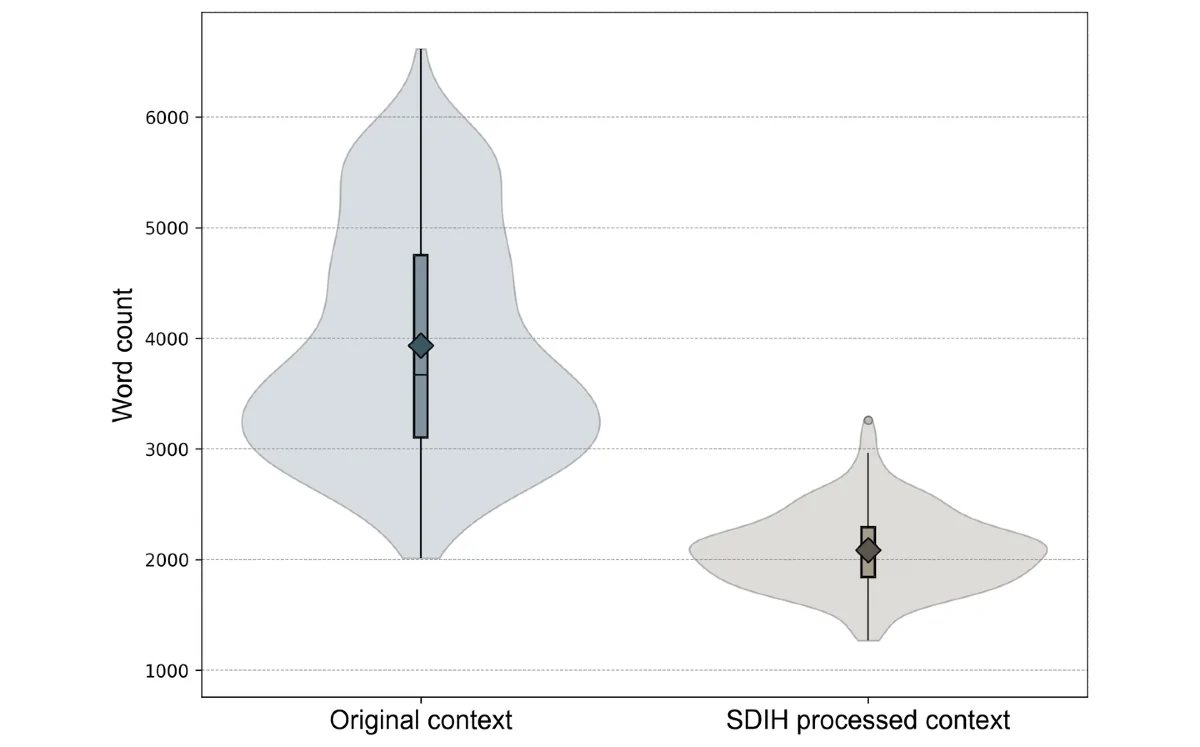
Distribution of health care context lengths before and after static dynamic information hierarchy (SDIH) module. The violin plots, with superimposed box plots, show the word count (y-axis) of the input health care context for the original (unprocessed) and SDIH-processed context (x-axis). Within each violin, the outer shape represents the kernel density distribution of case-level word counts, and the embedded box plot indicates the median (center line), IQR (box, first to third quartile), and whiskers extending to the most extreme values within 1.5 times the IQR; individual data points falling beyond the whiskers are plotted separately as outliers.

**Figure 4:**
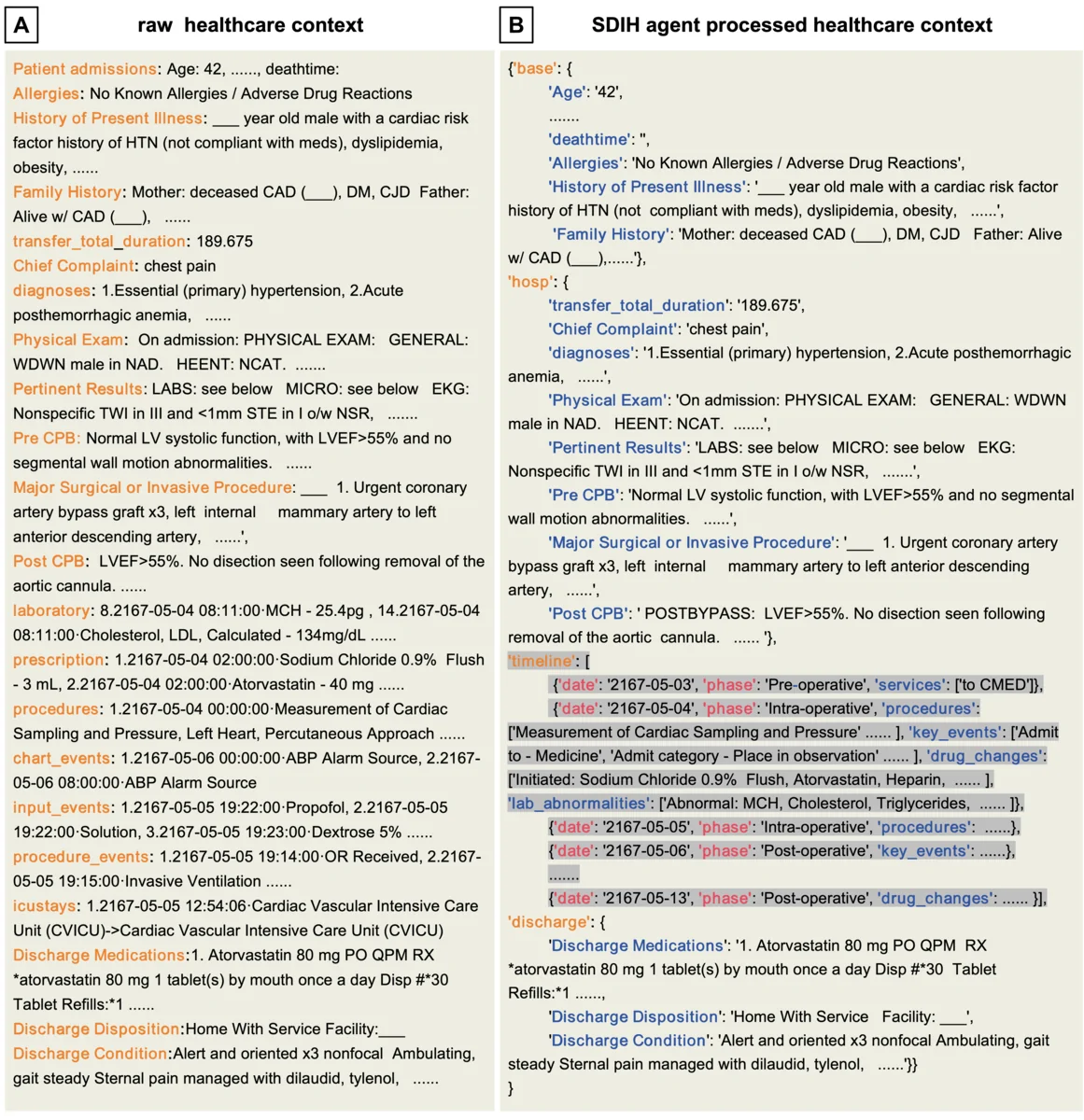
Example of original and processed input in a health care context. (A) Excerpt from an unprocessed (original) health care context for a coronary artery bypass grafting case. (B) The same case after applying the static dynamic information hierarchy module. Ellipses (“...”) indicate where lengthy repetitive text was truncated for display. Note: the data snippet presented in this figure is sourced from the publicly available, open-access Medical Information Mart for Intensive Care-IV-Demo dataset for illustrative purposes.

**Figure 5:**
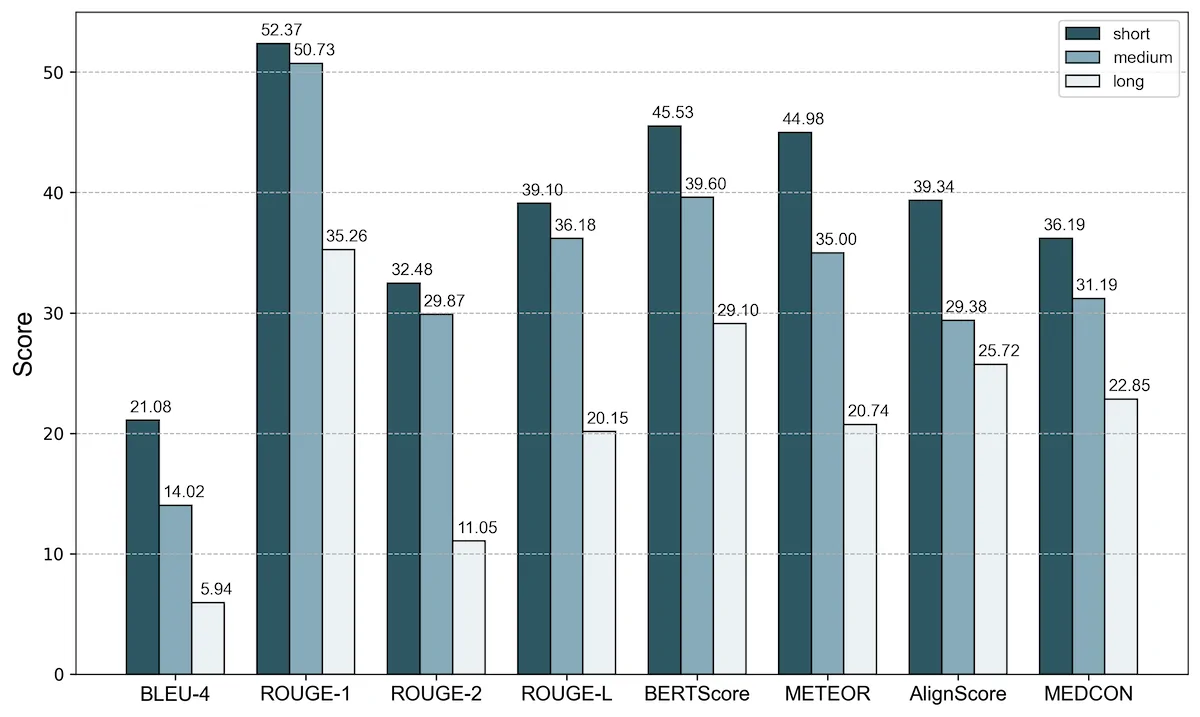
Summarization performance by length of input health care context. Cases were classified as short (≤ 3000 words: n=86), medium (>3000 to ≤5000 words: n=237), or long (>5000 words: n=80).

### Contribution of Each Module (Ablation Study)

#### Overview

To quantify the specific value of each component within our framework, we conducted an ablation study by progressively enabling the module on the 8B base LLM (Table 1 provides detailed results).

#### +SDIH Only

Structured condensation alone improved multiple metrics, with BLEU-4 increasing from 1.77 to 5.27, ROUGE-1 from 30.73 to 38.11, BERTScore from 20.50 to 30.88, and METEOR from 17.12 to 25.35. This 8B configuration markedly narrowed the gap with the raw 70B model, underscoring that a well-structured input substantially helps a smaller model.

#### +SD-RAG Only

Retrieval without prior condensation produced only marginal benefit (eg, ROUGE-1, BERTScore, METEOR, and MEDCON). Under this condition, adding similar-case text to an already lengthy unstructured input appeared to dilute, rather than focus, the clinically relevant signal.

#### +SDIH and SD-RAG

The combination of these 2 modules yielded a synergistic effect. Relative to either module alone, this configuration improved BLEU-4 to 12.80, ROUGE-2 to 13.16, BERTScore to 36.93, METEOR to 32.98, and MEDCON to 30.25. Once the input had been condensed into a concise chronological representation, retrieval became substantially more useful for recovering relevant details and clinical entities.

#### All Modules (+SDIH, SD-RAG, and SFO)

The addition of the SFO module provided the final refinement step, with further gains in ROUGE scores (ROUGE-1=52.78; ROUGE-2=28.81; and ROUGE-L=35.24 vs ROUGE-1=41.47; ROUGE-2=13.16; and ROUGE-L=22.34), modest increases in BERTScore and METEOR (38.67 and 34.20), improved factual alignment (AlignScore 30.78 vs 28.85), and preserved clinical concept coverage (MEDCON 30.41). These results suggest that the prompt refinements derived through the offline SFO process improved the final prompt configuration, reducing omissions and improving narrative fidelity.

### Cross-Domain Performance

Finally, we assessed the transferability under domain shift in a distinct clinical domain: lung lobectomy (thoracic surgery). The CABG-specific fine-tuned model did not show consistent improvement over the base model: relative to Llama 3.1-8B, it was higher on BLEU-4, ROUGE-2, ROUGE-L, and AlignScore, but lower on ROUGE-1, BERTScore, METEOR, and MEDCON. In contrast, LiteMedDoc remained clearly higher than both baselines across all 8 metrics. These preliminary, within the same database findings suggest that the modular design may be more resilient to domain shift than a conventionally fine-tuned static model. The modular design, including the SDIH, SD-RAG, and SFO components, may facilitate adaptation to a different clinical context through prompt and retrieval updates without additional model fine-tuning.

## Discussion

### Principal Results

#### Overview

This study demonstrates that high-quality, clinically accurate BHC summaries can be generated using a lightweight (8B parameter) LLM deployed locally without resource-intensive fine-tuning. Across automated evaluation, LiteMedDoc consistently outperformed untuned general-purpose baselines (including Mistral-7B-v0.3, Llama 3.1-8B/70B, Qwen3-14B, and GPT-4o-mini) on all 8 metrics and achieved the highest ROUGE-1/2/L scores among all evaluated systems. Compared with the CABG-specific Llama-8B-SFT baseline, the pattern was more nuanced: LiteMedDoc led on overlap-based metrics, whereas the fine-tuned model remained stronger on some semantic or source-grounded metrics.

Expert clinical evaluation by 15 cardiac surgeons confirmed the AI-generated summaries achieved mean scores ≥3.0 across all dimensions (completeness, correctness, readability, conciseness, and global quality) on a 5-point Likert scale, consistent with baseline acceptability for a clinician-in-the-loop drafting workflow. Reviewers specifically highlighted AI-generated summaries’ superior logical structure and conciseness. In the sampled qualitative audit, no POD discrepancies were identified in the AI-generated summaries, whereas 4 of the 10 physician reference notes contained such errors. In a subsequent cohort-level screening reaudit, 288 (6.35%) of 4538 physician reference summaries were flagged as containing potential POD inconsistencies, although this automated estimate is likely conservative.

The results suggest that architectural design can contribute as much as, or more than, raw model scale in this specific summarization task. Simply moving from Llama 3.1-8B to Llama 3.1-70B, or switching to Mistral-7B-v0.3, Qwen3-14B, or GPT-4o-mini, did not reproduce the gains achieved by LiteMedDoc. While raw LLMs often struggle with the “lost-in-the-middle” phenomenon when processing lengthy EHRs [42], our SDIH module effectively mimics expert clinical cognition by segmenting and compressing dynamic temporal data before generation. This decompose-and-conquer strategy allowed the 8B model to focus on narrative synthesis rather than on information extraction. Furthermore, in a preliminary within-database domain-shift test, the framework remained stronger than the CABG-specific fine-tuned model across lexical, semantic, and source-grounded metrics.

#### Clinical Application

The translation of AI summarization into practice hinges not just on linguistic fluency but also on factual precision. A notable finding from our temporal audit was that, in the sampled qualitative review, no POD discrepancies were identified in the AI-generated summaries. In contrast, such errors were present in a subset of physician reference notes. Although the magnitude of this cohort-level estimate should be interpreted cautiously, these findings collectively suggest that AI tools, when constrained by structured inputs (via the SDIH module), may help reduce certain documentation inconsistencies in clinician-in-the-loop workflows.

Integrating LiteMedDoc into the EHR workflow offers a dual benefit: efficiency and standardization. By automating the initial draft, clinicians can shift their role from “composer” to “editor,” potentially reclaiming hours of direct patient care time [4]. Moreover, the standardized output format could improve information continuity during care transitions, reducing the risk of readmissions caused by fragmented communication [43].

However, we emphasize that this is a “human-in-the-loop” system: the summaries are intended to assist, not replace clinicians [7,44]. Physicians must review and validate the AI-generated summaries as a safeguard against any errors or context nuances the AI might miss [8]. In practice, such human-in-the-loop AI documentation tools can reduce documentation time and improve note quality, combining the speed of AI with the expertise of a human provider [40]. This human-AI collaboration model is likely the safest and most effective way to implement the technology.

### Comparison With Prior Work

Current approaches to clinical summarization generally fall into 2 broad paradigms: closed-source systems and open-source models. LiteMedDoc was designed to address limitations in both paradigms.

The first paradigm relies on high-performance closed-source models accessed via an API [45,46]. Studies such as RUSSELL-GPT [20] and others [47] show that these models can achieve strong clinical summarization quality. In our benchmark, GPT-4o-mini served as a practical closed-source reference, yet LiteMedDoc exceeded it on all 8 automated metrics while remaining deployable entirely within local hospital infrastructure. This is particularly relevant for health care organizations in which privacy, governance, and integration requirements limit routine use of APIs.

The second paradigm involves adapting open-source models [17,48,49], but often requires additional computational resources, retraining, and maintenance and may show reduced robustness under domain shift. Our Llama-8B-SFT baseline showed that fine-tuning can improve selected metrics, but it also introduced clear domain brittleness on lobectomy. WisPerMed, another strong task-relevant benchmark, combined instruction tuning, additional clinical priming, and dynamic expert selection across multiple candidate models. Although it reported strong BERTScore, METEOR, and MEDCON, its published BHC results were obtained on a broader MIMIC-IV cohort, and its pipeline requires training and operating multiple models and selecting outputs per case. That level of GPU, engineering, and maintenance overhead is less aligned with typical hospital deployment needs, which often favor compact and locally maintainable systems.

In contrast, the main contribution of LiteMedDoc lies in demonstrating that a relatively small open-source model can be made clinically useful through a lightweight modular design rather than heavy retraining. The framework combines (1) structured temporal condensation of heterogeneous EHR inputs, (2) contextual-based retrieval, and (3) offline feedback-driven prompt optimization in a plug-and-play manner. This architecture offers practical advantages for hospital adoption, including local deployment, modular extensibility, lower coupling between components, and easier adaptation to new specialties through prompt or retrieval updates [50,51] rather than model retraining.

Furthermore, unlike “black-box” end-to-end generation methods, our modular pipeline offers interpretability and control. Recent multiagent frameworks [52,53] have attempted similar decompositions but often experienced error propagation. LiteMedDoc mitigates this disadvantage via the SFO module, which acts as an offline quality-assurance mechanism for prompt revision. This mirrors the “verify-and-edit” workflow proposed in Almanac [50] but applies it primarily during development and update cycles.

### Limitations

This study has certain limitations. First, evaluation was conducted using the MIMIC-IV database, which represents a single academic medical center. Although we included a within-database domain-shift in lobectomy cases, the test set was small (n=34) and was drawn from the same source database. Accordingly, the generalizability of LiteMedDoc to other institutions, community settings, and different documentation or EHR environments remains to be tested. Second, expert clinician evaluation was nonblinded to summary source, which may have introduced expectation bias in subjective ratings, although this design reflects real-world human-in-the-loop deployment. Moreover, the multireader review was necessarily restricted to a small set of cases (n=10). Consequently, the interrater reliability estimates were imprecise and were accompanied by wide CIs, and poor reliability could not be excluded for several domains. The degree of agreement among reviewers should therefore be regarded as preliminary rather than definitive, and larger-scale, multireader evaluations will be required to characterize it with confidence. Third, physician-authored discharge summaries were used as reference texts. Because these notes may themselves contain omissions or temporal inconsistencies, reference-based metrics may penalize model outputs. Our error analysis revealed that most low correctness ratings stemmed from apparent POD discrepancies, which resulted from inconsistencies in the physician reference notes. Meanwhile, the cohort-level POD reaudit relied on LLM-assisted screening rather than exhaustive human adjudication of the full reference corpus, so some inconsistencies were likely missed, and the reported 6.35% (288/4538) should be read as a conservative lower bound rather than the true error rate. This automated estimate is therefore not directly comparable with the higher rate observed in the small 10-case sample (4/10), whose divergence may reflect sampling variability, imperfect screening sensitivity, or both. Future work should validate this auditing approach against manually adjudicated samples. In addition, excluding extremely brief BHCs likely improved reference quality for retrieval and evaluation but may also have introduced selection bias. Although we manually reviewed the 29 excluded cases that, under the same random partitioning, would have fallen within the held-out test set, this subset represents less than 5% of the 646 excluded cases. This targeted review therefore cannot definitively rule out selection bias related to documentation style or patient complexity across the full excluded cohort. This exclusion may also have enriched the cohort for more standardized documentation patterns, leading to modest overestimation of performance relative to an unfiltered real-world population. Finally, generated summaries may still contain inaccuracies or oversimplified perioperative details. Because such errors could affect downstream referral or follow-up care if left uncorrected, LiteMedDoc should be used only within a human-in-the-loop workflow, with physician review required before any summary is finalized. Ongoing research and community collaboration are needed to further reduce hallucinations and improve reliability [54].

### Conclusions

In this study, we developed LiteMedDoc, a modular, locally deployable LLM-based framework for automated BHC summarization without model fine-tuning. By effectively decoupling knowledge retrieval and information organization from text generation, this modular approach overcomes the limitations of both cloud-based dependencies and rigid fine-tuning. In a retrospective evaluation on CABG cases from MIMIC-IV, LiteMedDoc demonstrated strong performance across automated metrics and achieved clinician ratings above the prespecified acceptability threshold for a clinician-in-the-loop drafting workflow. In a preliminary within-database cross-domain evaluation on lobectomy cases with prompt adaptation, LiteMedDoc maintained performance under domain shift compared with a CABG-fine-tuned baseline. These findings support the feasibility of privacy-preserving, resource-efficient summarization using a lightweight framework intended for clinician-in-the-loop deployment.

## Appendix

Multimedia Appendix 1 Technical implementation details of the similar document retrieval-augmented generation.

Multimedia Appendix 2 Technical implementation details of the SFO module’s iterative refinement loop.

Multimedia Appendix 3 The example of structure and instruction prompt.

Multimedia Appendix 4 Expert review questionnaire.

## Abbreviations

AlignScore: Alignment Score
BHC: brief hospital course
BioNLP: Biomedical Natural Language Processing
BLEU-4: Bilingual Evaluation Understudy-4 gram
CABG: coronary artery bypass grafting
EHR: electronic health record
FAISS: Facebook AI Similarity Search
ICC: intraclass correlation coefficient
ICD: International Classification of Diseases
LLM: large language model
LoRA: low-rank adaptation
MEDCON: Medical Concept Overlap
METEOR: Metric for Evaluation of Translation with Explicit Ordering
MIMIC-IV: Medical Information Mart for Intensive Care-IV
NLP: natural language processing
POD: postoperative day
ROUGE: Recall-Oriented Understudy for Gisting Evaluation
SDIH: static dynamic information hierarchy
SD-RAG: similar document retrieval-augmented generation
SFO: self-adaptive feedback optimization

## Data Availability

The data used in this study were derived from the publicly available Medical Information Mart for Intensive Care IV database, which is hosted on PhysioNet. Access to Medical Information Mart for Intensive Care IV requires completion of the Collaborative Institutional Training Initiative “Data or Specimens Only Research” course and acceptance of the PhysioNet Credentialed Health Data Use Agreement. Due to data use agreement restrictions and patient privacy considerations, the raw data cannot be shared directly. However, the datasets generated and analyzed during this study are reproducible using the described cohort extraction criteria. To further facilitate reproducibility, the cohort-construction and preprocessing scripts will be released in a public repository.

## References

[1] Shanafelt TD, Dyrbye LN, Sinsky C, Hasan O, Satele D, Sloan J, West CP (2016). Relationship between clerical burden and characteristics of the electronic environment with physician burnout and professional satisfaction. Mayo Clin Proc.

[2] Hao S (2023). Burnout and depression of medical staff: a chain mediating model of resilience and self-esteem. J Affect Disord.

[3] Bruyneel A, Bouckaert N, Maertens de Noordhout C, Detollenaere J, Kohn L, Pirson M, Sermeus W, Van den Heede K (2023). Association of burnout and intention-to-leave the profession with work environment: a nationwide cross-sectional study among Belgian intensive care nurses after two years of pandemic. Int J Nurs Stud.

[4] Sinsky C, Colligan L, Li L, Prgomet M, Reynolds S, Goeders L, Westbrook J, Tutty M, Blike G (2016). Allocation of physician time in ambulatory practice: a time and motion study in 4 specialties. Ann Intern Med.

[5] Tesfaye W, Jordan M, Chen TF, Castelino RL, Sud K, Dabliz R, Aslani P (2024). Usability evaluation methods used in electronic discharge summaries: literature review. J Med Internet Res.

[6] Kripalani S, LeFevre F, Phillips CO, Williams MV, Basaviah P, Baker DW (2007). Deficits in communication and information transfer between hospital-based and primary care physicians: implications for patient safety and continuity of care. JAMA.

[7] Tung JYM, Gill SR, Sng GGR, Lim DYZ, Ke Y, Tan TF, Jin L, Elangovan K, Ong JCL, Abdullah HR, Ting DSW, Chong TW (2024). Comparison of the quality of discharge letters written by large language models and junior clinicians: single-blinded study. J Med Internet Res.

[8] Williams CYK, Subramanian CR, Ali SS, Apolinario M, Askin E, Barish P, Cheng M, Deardorff WJ, Donthi N, Ganeshan S, Huang O, Kantor MA, Lai AR, Manchanda A, Moore KA, Muniyappa AN, Nair G, Patel PP, Santhosh L, Schneider S, Torres S, Yukawa M, Hubbard CC, Rosner BI (2025). Physician- and large language model-generated hospital discharge summaries. JAMA Intern Med.

[9] Syversen MO, Glatkauskas M, Sedeniussen SJ, Hauge M, Benny S, Horgen K, Lea M, Romskaug R, Njaastad AM, Wibe T, Svensberg K, Mathiesen L (2025). Discrepancies in medication lists after hospital discharge in patients with multiple long-term conditions. Res Social Adm Pharm.

[10] Liu J, Wang C, Liu S (2023). Utility of ChatGPT in clinical practice. J Med Internet Res.

[11] Searle T, Ibrahim Z, Teo J, Dobson RJ (2023). Discharge summary hospital course summarisation of in patient electronic health record text with clinical concept guided deep pre-trained transformer models. J Biomed Inform.

[12] Scott D, Hallett C, Fettiplace R (2013). Data-to-text summarisation of patient records: using computer-generated summaries to access patient histories. Patient Educ Couns.

[13] Pivovarov R, Elhadad N (2015). Automated methods for the summarization of electronic health records. J Am Med Inform Assoc.

[14] Silver AM, Goodman LA, Burton M, Rangan P, Chadha R, Thomas AK, O'Donnell C (2026). Optimizing discharge summaries: a survey of inpatient clinician perspectives and the path to standardization. J Gen Intern Med.

[15] Ando K, Okumura T, Komachi M, Horiguchi H, Matsumoto Y (2022). Is artificial intelligence capable of generating hospital discharge summaries from inpatient records?. PLOS Digit Health.

[16] Singh S, Djalilian A, Ali MJ (2023). ChatGPT and ophthalmology: exploring its potential with discharge summaries and operative notes. Semin Ophthalmol.

[17] Bednarczyk L, Reichenpfader D, Gaudet-Blavignac C, Ette AK, Zaghir J, Zheng Y, Bensahla A, Bjelogrlic M, Lovis C (2025). Scientific evidence for clinical text summarization using large language models: scoping review. J Med Internet Res.

[18] Schwieger A, Angst K, de Bardeci M, Burrer A, Cathomas F, Ferrea S, Grätz F, Knorr M, Kronenberg G, Spiller T, Troi D, Seifritz E, Weber S, Olbrich S (2024). Large language models can support generation of standardized discharge summaries - a retrospective study utilizing ChatGPT-4 and electronic health records. Int J Med Inform.

[19] Barak-Corren Y, Wolf R, Rozenblum R, Creedon JK, Lipsett SC, Lyons TW, Michelson KA, Miller KA, Shapiro D, Reis BY, Fine AM (2024). Harnessing the power of generative AI for clinical summaries: perspectives from emergency physicians. Ann Emerg Med.

[20] Chua CE, Lee Ying Clara N, Furqan MS, Lee Wai Kit J, Makmur A, Tham YC, Santosa A, Ngiam KY (2024). Integration of customised LLM for discharge summary generation in real-world clinical settings: a pilot study on RUSSELL GPT. Lancet Reg Health West Pac.

[21] Goswami J, Prajapati Kk, Saha A, Saha Ak (2024). Parameter-efficient fine-tuning large language model approach for hospital discharge paper summarization. Appl Soft Comput.

[22] Xu J Discharge me: BioNLP ACL-24 shared task on streamlining discharge documentation. Physionet.org.

[23] Patel SB, Lam K (2023). ChatGPT: the future of discharge summaries?. Lancet Digit Health.

[24] Ganzinger M, Kunz N, Fuchs P, Lyu CK, Loos M, Dugas M, Pausch TM (2025). Automated generation of discharge summaries: leveraging large language models with clinical data. Sci Rep.

[25] Johnson AEW, Bulgarelli L, Shen L, Gayles A, Shammout A, Horng S, Pollard TJ, Hao S, Moody B, Gow B, Lehman LH, Celi LA, Mark RG (2023). MIMIC-IV, a freely accessible electronic health record dataset. Sci Data.

[26] Guo R, Farnan G, McLaughlin N, Devereux B (2024). QUB-Cirdan at “Discharge Me!”: zero shot discharge letter generation by open-source LLM. https://aclanthology.org/2024.bionlp-1.58.pdf.

[27] Dubey A, Jauhri A, Pandey A (2024). The Llama 3 herd of models. arXiv. Preprint posted online on July 31, 2024.

[28] Lewis P, Perez E, Piktus A (2020). Retrieval-augmented generation for knowledge-intensive NLP tasks. https://proceedings.neurips.cc/paper/2020/file/6b493230205f780e1bc26945df7481e5-Paper.pdf.

[29] Wang W, Wei F, Dong L, Bao H, Yang N, Zhou M (2020). MINILM: deep self-attention distillation for task-agnostic compression of pre-trained transformers. https://proceedings.neurips.cc/paper/2020/file/3f5ee243547dee91fbd053c1c4a845aa-Paper.pdf.

[30] Breiman L (2001). Random forests. Mach Learn.

[31] Jiang AQ, Sablayrolles A, Mensch A, Bamford C (2023). Mistral 7B. arXiv. Preprint posted online on October 10, 2023.

[32] Yang A, Li A, Yang B, Zhang B (2025). Qwen3 technical report. arXiv. Preprint posted online on May 14, 2025.

[33] (2024). GPT-4o mini: advancing cost-efficient intelligence. OpenAI.

[34] Papineni K, Roukos S, Ward T, Zhu WJ (2002). BLEU: a method for automatic evaluation of machine translation. https://aclanthology.org/P02-1040.pdf.

[35] Lin CY (2004). ROUGE: a package for automatic evaluation of summaries. https://aclanthology.org/W04-1013/.

[36] Zhang T, Kishore V, Wu F, Weinberger K (2020). BERTScore: evaluating text generation with BERT. OpenReview.

[37] Banerjee S, Lavie A (2005). METEOR: an automatic metric for MT evaluation with improved correlation with human judgments. https://aclanthology.org/W05-0909.pdf.

[38] Zha Y, Yang Y, Li R, Hu Z (2023). ALIGNSCORE: evaluating factual consistency with a unified alignment function. https://aclanthology.org/2023.acl-long.634.pdf.

[39] Yim W, Fu Y, Ben Abacha A, Snider N, Lin T, Yetisgen M (2023). Aci-bench: a novel ambient clinical intelligence dataset for benchmarking automatic visit note generation. Sci Data.

[40] Hartman V, Zhang X, Poddar R, McCarty M, Fortenko A, Sholle E, Sharma R, Campion T, Steel PAD (2024). Developing and evaluating large language model-generated emergency medicine handoff notes. JAMA Netw Open.

[41] Damm H, Pakull T, Eryılmaz B (2024). WisPerMed at “Discharge Me!”: advancing text generation in healthcare with large language models, dynamic expert selection, and priming techniques on MIMIC-IV. https://aclanthology.org/2024.bionlp-1.9.pdf.

[42] Landman AB, Tilak SS, Walker GA (2024). Artificial intelligence-generated emergency department summaries and hospital handoffs. JAMA Netw Open.

[43] Dharmarajan K, Wang Y, Lin Z, Normand ST, Ross JS, Horwitz LI, Desai NR, Suter LG, Drye EE, Bernheim SM, Krumholz HM (2017). Association of changing hospital readmission rates with mortality rates after hospital discharge. JAMA.

[44] Small WR, Austrian J, O'Donnell L, Burk-Rafel J, Hochman KA, Goodman A, Zaretsky J, Martin J, Johnson S, Major VJ, Jones S, Henke C, Verplanke B, Osso J, Larson I, Saxena A, Mednick A, Simonis C, Han J, Kesari R, Wu X, Heery L, Desel T, Baskharoun S, Figman N, Farooq U, Shah K, Jahan N, Kim JM, Testa P, Feldman J (2025). Evaluating hospital course summarization by an electronic health record-based large language model. JAMA Netw Open.

[45] Sivarajkumar S, Kelley M, Samolyk-Mazzanti A, Visweswaran S, Wang Y (2024). An empirical evaluation of prompting strategies for large language models in zero-shot clinical natural language processing: algorithm development and validation study. JMIR Med Inform.

[46] Ralevski A, Taiyab N, Nossal M, Mico L, Piekos S, Hadlock J (2024). Using large language models to abstract complex social determinants of health from original and deidentified medical notes: development and validation study. J Med Internet Res.

[47] Singhal K, Azizi S, Tu T, Mahdavi SS, Wei J, Chung HW, Scales N, Tanwani A, Cole-Lewis H, Pfohl S, Payne P, Seneviratne M, Gamble P, Kelly C, Babiker A, Schärli N, Chowdhery A, Mansfield P, Demner-Fushman D, Agüera Y Arcas B, Webster D, Corrado GS, Matias Y, Chou K, Gottweis J, Tomasev N, Liu Y, Rajkomar A, Barral J, Semturs C, Karthikesalingam A, Natarajan V (2023). Large language models encode clinical knowledge. Nature.

[48] Afzal M, Alam F, Malik KM, Malik GM (2020). Clinical context-aware biomedical text summarization using deep neural network: resentation model development and validation. J Med Internet Res.

[49] Cho HN, Jun TJ, Kim Y, Kang H, Ahn I, Gwon H, Kim Y, Seo J, Choi H, Kim M, Han J, Kee G, Park S, Ko S (2024). Task-specific transformer-based language models in health care: scoping review. JMIR Med Inform.

[50] Zakka C, Shad R, Chaurasia A, Dalal AR, Kim JL, Moor M, Fong R, Phillips C, Alexander K, Ashley E, Boyd J, Boyd K, Hirsch K, Langlotz C, Lee R, Melia J, Nelson J, Sallam K, Tullis S, Vogelsong MA, Cunningham JP, Hiesinger W (2024). Almanac - retrieval-augmented language models for clinical medicine. NEJM AI.

[51] Miao Y, Zhao Y, Luo Y, Wang H, Wu Y (2025). Improving large language model applications in the medical and nursing domains with retrieval-augmented generation: scoping review. J Med Internet Res.

[52] Borkowski AA, Ben-Ari A (2025). Multiagent AI systems in health care: envisioning next-generation intelligence. Fed Pract.

[53] Mehandru N, Miao BY, Almaraz ER, Sushil M, Butte AJ, Alaa A (2024). Evaluating large language models as agents in the clinic. NPJ Digit Med.

[54] Raghu Subramanian C, Rosner BI (2025). Advancing toward clinical deployment of AI-generated discharge summaries-beyond the bench. JAMA Netw Open.

