# Supplementary material for: Automated Brief Hospital Course Summarization in Cardiac Surgery Using a Lightweight Large Language Model–Based Framework: Development and Evaluation Study on the Medical Information Mart for Intensive Care-IV
Source: J Med Internet Res. 2026 Sep 24;28:e90870. doi: 10.2196/90870 (PMC13650456; doi:10.2196/90870)
Supplement: Supplementary file 1 [file jmir_v28i1e90870_app1.docx]

**Multimedia Appendix 1: Technical Implementation Details of the SD-RAG.**

The SD-RAG module represents all 4,135 reference BHC summaries as vector embeddings using a MiniLM sentence encoder, and it indexes these embeddings with FAISS for fast similarity search. The module prioritizes five key patient features (identified via feature importance analysis [29]) to enhance clinically relevant matching: hospital length of stay, notable discharge exam findings, discharge condition, major procedures during hospitalization, and significant lab results.

Because the retrieval features of hospital length of stay and discharge condition are only finalized at discharge, their usage depends on the drafting time point. In our retrospective evaluation, these fields were obtained from finalized discharge documentation in MIMIC-IV and were therefore available at generation time. In prospective workflows where clinicians begin drafting earlier, contemporaneously documented fields (eg, the expected discharge date and anticipated discharge disposition, which are typically updated longitudinally during hospitalization) can be used to generate an initial draft, which can be readily regenerated once final discharge values are confirmed.

Using a composite score of embedding similarity and feature overlap, the SD-RAG module retrieves the top three most similar cases from the library. The BHC sections of these retrieved cases are appended to the LLM’s input prompt to augment the context with real examples.
