# Supplementary material for: Automated Brief Hospital Course Summarization in Cardiac Surgery Using a Lightweight Large Language Model–Based Framework: Development and Evaluation Study on the Medical Information Mart for Intensive Care-IV
Source: J Med Internet Res. 2026 Sep 24;28:e90870. doi: 10.2196/90870 (PMC13650456; doi:10.2196/90870)
Supplement: Supplementary file 2 [file jmir_v28i1e90870_app2.docx]

## Multimedia Appendix 2: Technical Implementation Details of the SFO Module’s Offline Prompt-Optimization Workflow.

The SFO module’s iterative refinement loop is as follows:

1. Draft Generation: For a batch of cases, the module prompts the LLM to produce BHC drafts from the structured input and retrieved examples using the prevailing prompt.

2. Quality Evaluation: The drafts are reviewed for completeness, chronological accuracy, terminology correctness, and proper de-identification.

3. Feedback Generation: When deficiencies are identified, feedback instructions detailing the required improvements are composed from the qualitative review.

4. Prompt Revision: The prevailing prompt is revised according to the feedback instructions to yield an updated configuration.

5. Validation of the Revised Prompt: New drafts are generated for the batch under the updated prompt and scored on the eight automated metrics.

6. Batch Progression: The updated prompt is carried forward to the next batch, and the procedure is repeated.

In this study, the SFO loop was applied offline with 50 cases per batch. For each batch, drafts were generated with the inherited prompt and reviewed across the four quality dimensions to synthesize feedback. After qualitative feedback from a development batch had been used to revise the prompt, the candidate prompt was re-evaluated on that same development batch using all eight automated metrics. A candidate prompt replaced the currently retained configuration only if it improved more than half of the eight metrics; optimization was terminated after three consecutive batches failed to meet this retention criterion or when all available development cases had been processed.

During offline optimization, predefined metric thresholds (ROUGE-1 >40, ROUGE-2 >20, and ROUGE-L >30; BLEU-4, BERTScore, METEOR, AlignScore, and MEDCON targets were defined relative to the corresponding Llama 3.1-70B values) were used as reference targets for monitoring optimization progress. Prompt refinement was performed iteratively across evaluation batches.

In this study, a development set of 400 cases (eight batches of 50 cases) was prepared, of which 300 (six batches) were processed before early stopping (Table S1). Batch 3 satisfied the predefined retention criterion and replaced the previous configuration. The subsequent three batches (batches 4-6) did not meet the retention criterion because improvements were limited to a minority of the evaluated metrics. After three consecutive nonqualifying batches, optimization was terminated according to the early-stopping rule, and the batch 3 prompt configuration was retained for subsequent inference.

Table S1. Batch-wise trajectory of offline prompt optimization in the SFO module.

| Batch (cases) | BLEU-4 | ROUGE-1 | ROUGE-2 | ROUGE-L | BERTScore | METEOR | AlignScore | MEDCON | action |
| --- | --- | --- | --- | --- | --- | --- | --- | --- | --- |
| 1 (1–50) | 10.93 | 42.63 | 16.08 | 24.11 | 38.56 | 33.90 | 26.23 | 27.68 | continue |
| 2 (51–100) | 11.41 | 47.05 | 14.80 | 25.66 | 38.68 | 33.52 | 26.34 | 24.93 | continue |
| 3 (101–150) | 13.07 | 50.69 | 24.77 | 28.20 | 40.37 | 33.72 | 25.38 | 28.49 | retained |
| 4 (151–200) | 12.44 | 45.86 | 21.86 | 29.07 | 39.24 | 33.49 | 26.72 | 25.64 | patience |
| 5 (201–250) | 9.17 | 46.05 | 15.71 | 24.29 | 35.09 | 34.97 | 21.56 | 25.77 | patience |
| 6 (251–300) | 8.43 | 41.50 | 14.03 | 24.12 | 33.05 | 28.32 | 25.73 | 24.74 | stop |

This offline feedback mechanism refines the prompt configuration across batches without any human intervention and without updating its learned parameters. As a result, the module-based framework remains lightweight. To adapt the system to a new domain or document style, one can adjust the prompts or feedback rules rather than retrain the model, making this approach highly adaptable to different clinical settings.

In routine deployment, LiteMedDoc generates a single clinician-editable draft and does not require per-patient iterative optimization. The SFO module is primarily used in three scenarios: (a) offline prompt optimization using previously completed cases, (b) updates when documentation requirements change during deployment, and (c) prompt adaptation when transferring to a new domain or institution. Reference-based metrics (e.g., BLEU and ROUGE) are computed retrospectively for benchmarking when a reference summary exists but are not used as real-time stopping criteria for new patients.
