# Supplementary material for: Automated Brief Hospital Course Summarization in Cardiac Surgery Using a Lightweight Large Language Model–Based Framework: Development and Evaluation Study on the Medical Information Mart for Intensive Care-IV
Source: J Med Internet Res. 2026 Sep 24;28:e90870. doi: 10.2196/90870 (PMC13650456; doi:10.2196/90870)
Supplement: Supplementary file 3 [file jmir_v28i1e90870_app3.docx]

## Multimedia Appendix 3: The Example of Structure and Instruction Prompt.

Figure S1. Illustration of “structure prompt”.

Figure S2. Illustration of “instruction prompt”.
