# Supplementary material for: Automated Brief Hospital Course Summarization in Cardiac Surgery Using a Lightweight Large Language Model–Based Framework: Development and Evaluation Study on the Medical Information Mart for Intensive Care-IV
Source: J Med Internet Res. 2026 Sep 24;28:e90870. doi: 10.2196/90870 (PMC13650456; doi:10.2196/90870)
Supplement: Supplementary file 4 [file jmir_v28i1e90870_app4.docx]

## Multimedia Appendix 4: Expert Review Questionnaire.

Please evaluate the generated medical note in comparison to the reference (actual) note across the following dimensions:

1. Completeness (captures important information):

To what extent does the generated note include key clinical concepts, findings, and events present in the reference note?

- Captures no important information (1)
- Captures ~25% of the important information (2)
- Captures ~50% of the important information (3)
- Captures ~75% of the important information (4)
- Captures all of the important information (5)

2. Correctness (contains less false information):

Does the generated note contain any incorrect or misleading clinical information? Consider potential impacts on patient care

- Contains harmful content that will definitely impact future care (1)
- Contains incorrect content that is likely to impact future care (2)
- Contains incorrect content that may or may not impact future care (3)
- Contains incorrect content that will not impact future care (4)
- Contains no incorrect content (5)

3. Readability (Clarity and Structure):

How readable and logically structured is the generated note compared to the reference note?

- Significantly harder to read than the reference text (1)
- Slightly harder to read than the reference text (2)
- Neither easier nor harder to read than the reference text (3)
- Slightly easier to read than the reference text (4)
- Significantly easier to read than the reference text (5)

4. Conciseness (less non-important information):

Does the generated note contain any unnecessary or redundant clinical information?

- Contains excessive non-important or redundant information (1)
- Contains a lot of non-important information (2)
- Includes some non-important information, but still usable (3)
- Mostly concise, with minimal non-important information (4)
- Highly concise with only relevant, meaningful information (5)

5. Global (Holistic Comparison to the Reference Text):

Considering completeness, correctness, readability, and conciseness, how does the generated note compare to the reference note in terms of overall quality?

- Significantly worse than the reference text (1)
- Slightly worse than the reference text (2)
- Neither better nor worse than the reference text (3)
- Slightly better than the reference text (4)
- Significantly better than the reference text (5)
